# Comparison of the effectiveness of the levonorgestrel-intrauterine device and oral progestogens on regression of endometrial hyperplasia without atypia

**DOI:** 10.1016/j.heliyon.2022.e12150

**Published:** 2022-12-07

**Authors:** Ye Shen, Hua Fang, Yi Zhang, Yan Du, Rong Cai, Min Zhao, Qi Chen

**Affiliations:** a Department of Family Planning, Wuxi Maternity and Child Health Hospital Affiliated Nanjing Medical University, Wuxi, China; b Department of Obstetrics & Gynaecology, The University of Auckland, Auckland, New Zealand; c Department of Pathology, Wuxi Maternity and Child Health Hospital Affiliated Nanjing Medical University, Wuxi, China; d Department of Ultrasound, Wuxi Maternity and Child Health Hospital Affiliated Nanjing Medical University, Wuxi, China; e Department of Gynaecology, Wuxi Maternity and Child Health Hospital Affiliated Nanjing Medical University, Wuxi, China

**Keywords:** Endometrial hyperplasia, LNG-IUD, Oral progestogens, Regression, Follow-up

## Abstract

Endometrial hyperplasia is caused by an excess of estrogen unopposed by progesterone. Oral progestogens are traditionally used for endometrial hyperplasia without atypia. However oral progestogen is not always successful at causing regression of endometrial hyperplasia. In addition, cyclic progestogens are less effective in delivering progestogen to the endometrium. Therefore, the levonorgestrel-intrauterine device (LNG-IUD), as an alternative option of delivery progestogen has been introduced in clinical practice. The effectiveness of LNG-IUD in causing regression of endometrial hyperplasia in the short-term had moderate-quality evidence, but the long-term (13 months to two years) effectiveness had low-quality evidence. In this study with relatively large sample size, we compared the effectiveness in the regression of endometrial hyperplasia without atypia for short-term and long-term between the treatment with LNG-IUD and oral progestogens or no treatment. Data on histology or ultrasound from 466 cases who received either LNG-IUD or oral progestogens or were untreated were collected. The primary treatment with LNG-IUD showed a 93% regression rate of endometrial hyperplasia, which was significantly higher than oral progestogens showing a 66% regression rate. The odds ratio of regression of endometrial hyperplasia in cases with LNG-IUD treatment was 7.128 (95%CI: 2.94, 16.76, p < 0.0001), compared to the cases with oral progestogen treatment. The regression rate in untreated cases was 16%. In addition, cases without regression by oral progestogens who then received the alternative treatment option by LNG-IUD also showed a 93% regression rate. While continuously receiving oral progestogens showed a 55% regression rate of endometrial hyperplasia, which was significantly lower than LNG-IUD treatment as an alternative option. Our data reports a significant response on regression of endometrial hyperplasia after LNG-IUD treatment in comparison with oral progestogen treatment.

## 1 Introduction

Endometrial hyperplasia is an overgrowth of epithelial cells in the endometrium resulting from an excess of estrogen, in which the uterine lining becomes too thick and causes abnormal vaginal bleeding. The estimated incidence of endometrial hyperplasia without atypia was 1–2 women per thousand women in the USA [1]. Endometrial hyperplasia could have some risk factors for developing endometrial carcinoma, as recent studies showed that in certain women, endometrial hyperplasia significantly increases the risk of developing endometrial cancer later [2, 3].

The options of treatment for endometrial hyperplasia depend on the subtypes of endometrial hyperplasia, but oral progestogens, including megestrol acetate and medroxyprogesterone, are traditionally used for the treatment of endometrial hyperplasia without atypia [4, 5]. This is because that endometrial hyperplasia without atypia has a lower risk of progression to atypia or cancer, compared to other types of endometrial hyperplasia [4]. However, oral progestogens are not always effective at causing regression of endometrial hyperplasia and may have some side effects [6]. Levonorgestrel-intrauterine device (LNG-IUD), as an alternative option for delivery progestogens has been introduced in clinical practice for the treatment of endometrial hyperplasia, despite a lack of strong evidence on the effectiveness of LNG-IUD in regression of the hyperplasia [7, 8].

LNG-IUD can directly deliver progestogens into the endometrial cavity without the adverse effects of systemic progestogens [9]. Unopposed estrogen is the most common risk factor for developing endometrial hyperplasia and endometrial cancer by promoting the growth of all endometrial cell types [10]. Progestogens are believed to terminally differentiate epithelium, induce apoptosis, and prevent estrogen-induced proliferation [11, 12, 13, 14]. A systematic review and meta-analysis with 24 observational studies reported a lower disease regression rate by oral progestogens, compared to LNG-IUD in the treatment of endometrial hyperplasia [15]. Another meta-analysis study with 7 randomised controlled trials (RCTs) reported a higher regression rate by LNG-IUD in the treatment of endometrial hyperplasia without atypia [16]. Including a recent Cochrane study with 11 RCTs [6], these studies clearly suggested a short-term (within 12 months) effectiveness of regression with the treatment of LNG-IUD in endometrial hyperplasia, while the long-term (longer than 13 months) effectiveness of LNG-IUD in the treatment of endometrial hyperplasia is less well described in the literature.

Therefore, the objectives of this retrospective study with a relatively large sample size are 1) to compare the effectiveness in the regression of endometrial hyperplasia without atypia in the short-term (within 12 months) between the treatment with LNG-IUD and oral progestogens or no treatment; 2) to analyse the long-term (longer than 13 months) effectiveness in the regression of endometrial hyperplasia without atypia by the treatment with LNG-IUD; 3) to investigate the effectiveness of regression in cases who failed with the primary treatment of oral progestogens and then received LNG-IUD as the secondary treatment option.

## 2 Methods

This retrospective study received ethics approval by the Ethics Committee of Wuxi Maternity and Child Health Hospital affiliated Nanjing Medical University, Wuxi, China (reference number: 2021/06/1112-24). The consent form from patients was not required for this retrospective study.

### 2.1 Study cohort

**Figure 1:**
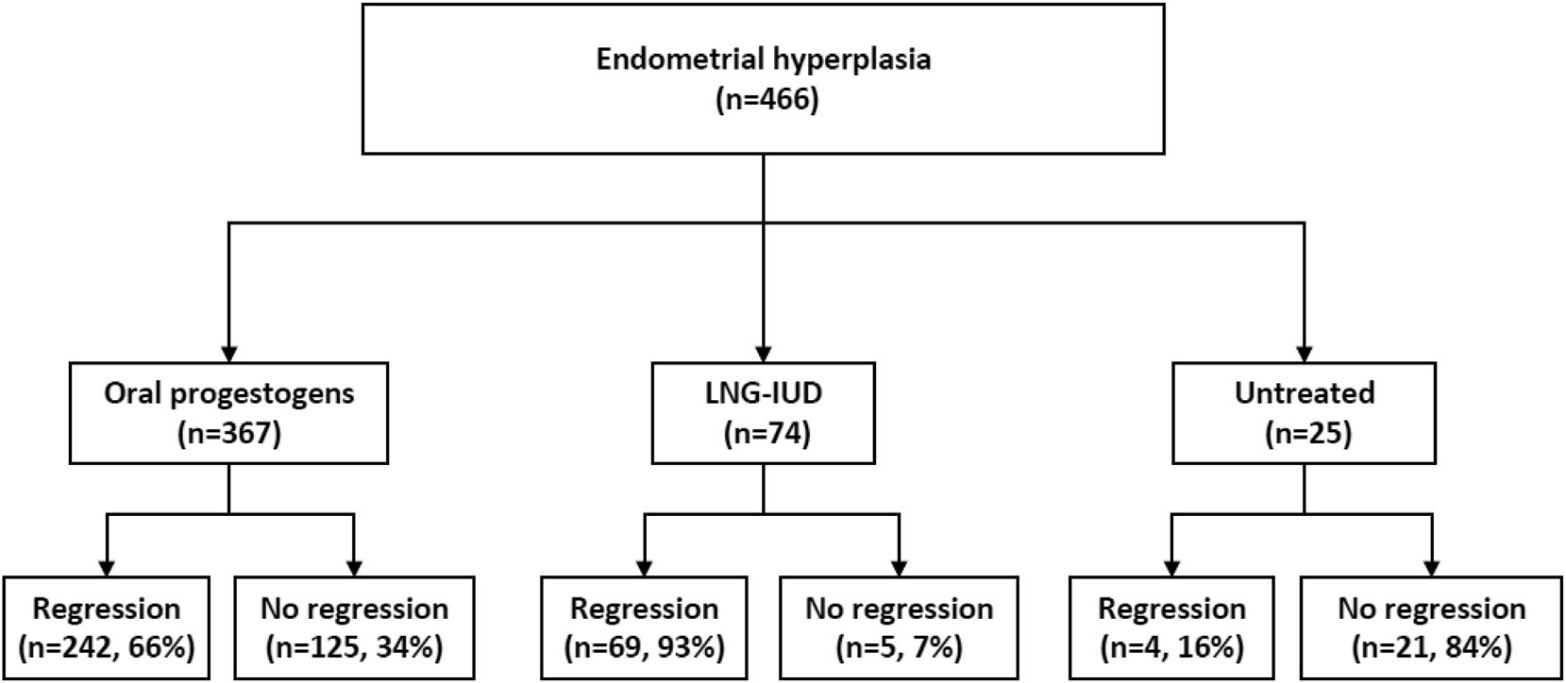
Clinical outcomes in the primary treatment.

**Figure 2:**
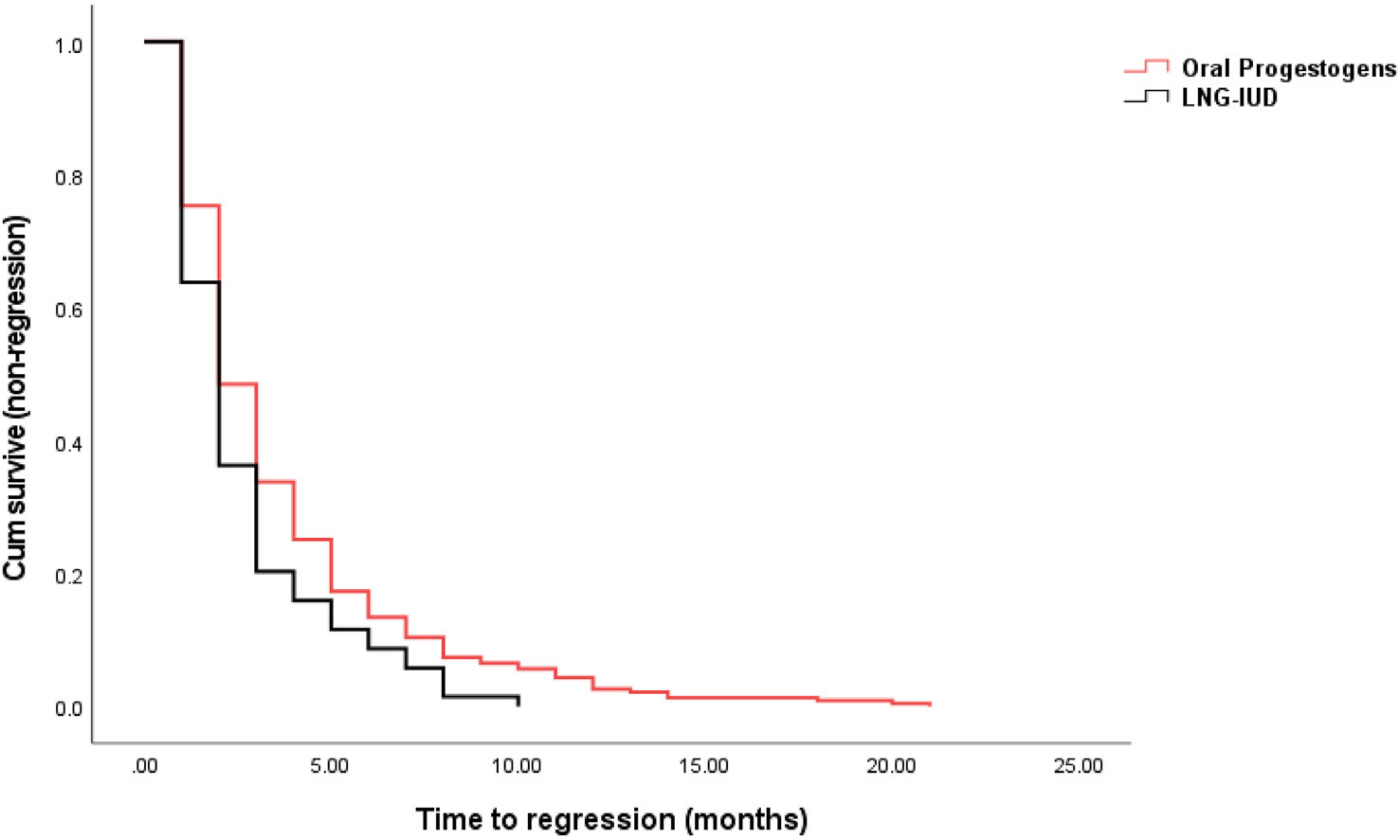
Kaplan-Meier curve showing the time to regression between the two groups.

From January 2015 to March 2021, a total number of 466 women who were newly diagnosed with endometrial hyperplasia without atypia from our hospital database were included in this study. All the cases did not receive any previous treatment. The diagnosis of endometrial hyperplasia was confirmed by endometrial biopsy. Of these cases, 441 cases primarily received the treatment with either oral progestogens or LNG-IUD, while 25 cases did not receive any treatment for at least three months (Figure 1). 108 cases who failed the primary treatment with oral progestogens received with LNG-IUD as an alternative option of treatment (referred to as secondary treatment), or continuously received the oral progestogens (Figure 2). The oral progestogens included megestrol acetate (40 mg/d), dydrogesterone (20 mg/d), fulepian (15 mg/d), enantone (3.75 mg/d) and progesterone soft cxapsules (250 mg/d). The criteria for the options of treatment between oral progestogens and LNG-IUD were based on individual cases and the experiences of individual gynaecologists. All the cases were followed up for more than 3 months, and the regression of endometrial hyperplasia was confirmed by histological or ultrasound diagnosis after the primary and secondary treatment. Data on histological or ultrasound diagnosis were collected from the hospital's electronic database.

Regression of endometrial hyperplasia, referred to as the effectiveness of treatment was defined as the absence of any evidence of endometrial hyperplasia, and no regression of endometrial hyperplasia was defined as no change from the histological or ultrasound diagnosis at baseline after the primary or secondary treatment. Progression of endometrial hyperplasia was defined for patients with endometrial hyperplasia if they progressed to endometrial hyperplasia with atypia or endometrial cancer from baseline.

The regression rate of endometrial hyperplasia, as the outcome of the primary treatment was analysed between cases who received oral progestogens and cases who received LNG-IUD. In addition, the regression rate of endometrial hyperplasia was also analysed in cases who failed with oral progestogens as the primary treatment and then received a different secondary treatment.

### 2.2 Statistical analysis

The age at diagnosis was expressed as mean and standard deviation (SD), as the age was normally distributed in our study cohort. The statistical difference in proportion of cases with regression or no regression or hysterectomy was assessed by Chi square test using GraphPad Prison (version 9.2). Time to regression was expressed as median and range. The statistical difference was assessed by Mann-Whitney U test, using GraphPad Prison (version 9.2). The odds ratio (OR) and 95% confidence intervals (CI) for the association of regression rate and treatment options were performed using GraphPad Prism (version 9.3), GraphPad Software, La Jolla, CA. p < 0.05 was considered as the threshold for statistical significance.

## 3 Results

A total of 466 cases with endometrial hyperplasia without atypia were included in this study. Of them, 367 cases primarily received treatment with at least one type of oral progestogens, and 74 cases primarily received treatment with LNG-IUD, and 25 cases did not receive any treatment at least for three months. The median age of women who were diagnosed with endometrial hyperplasia was 45 years (ranging from 23 to 60 years). The mean age of women who received oral progestogens, or LNG-IUD or untreated was 44.3 ± 6.5, or 44.3 ± 5.9, or 45.3 ± 7.3 years, respectively, which was not statistically different (p = 0.726, ANONA Kruskal-Wallis test) (Table 1). There was no data on menopause status, we then analysed the age difference between under and over 50 years among the three groups (Table 1). There was no difference in the proportion of women aged under and over 50 years among the three groups (p = 0.617).

**Table 1:** Clinical parameters in study cohort (n = 466).

	Untreated (n = 25)	Oral progestogens (n = 367)	LNG-IUD (n = 74)	P value
Age (years, median/range)	45 (29–60)	45 (23–59)	44 (25–56)	P = 0.272 (ANOVA)
Age <50 years (n, %)	19 (76%)	298 (81%)	60 (81%)	P = 0.617
Age ≥50 years (n, %)	6 (24%)	69 (19%)	14 (19%)	P = 0.814

In cases with oral progestogens as the primary treatment (n = 367), the regression rate of endometrial hyperplasia was 66% (242 out of 367) with a median of 10 months follow-up (3–68 months). In the group with LNG-IUD as the primary treatment (n = 74), the regression rate of endometrial hyperplasia was 93% (69 out of 74) with a median of 10 months follow-up (3–76 months) (Table 2). The odds ratio of regression of endometrial hyperplasia in cases after LNG-IUD treatment was 7.128 (95% CI: 2.94, 16.76, p < 0.0001), compared with the cases with oral progestogen treatment. In addition, we compared the time to regression between the two groups. As shown in Figure 2, the median time to regression in LNG-IUD treatment group was 2 months (ranging from 1 to 10 months), which was significantly lower than that in oral progestogen treatment group (2 months, ranging from 1 to 21 months, p = 0.022, Mann-Whitney U test).

**Table 2:** Clinical outcomes of primary treatments.

	Oral progestogens (n = 367)	LNG-IUD (n = 74)	OR (95% CI)∗	P value
Regression	242 (66%)	69 (93%)	7.128 (2.94, 16.76)	P < 0.0001
Non regression and progression	125 (34%)	5 (7%)		

∗ The odds ratio (OR) was calculated to compare the regression and non-regression and progression between the two treatments.

In comparison with women who either primarily received oral progestogens or LNG-IUD treatment, 25 cases did not receive any treatment for at least three months. Of them, only 4 (16%) cases had regression within 3 months, and 21 (84%) cases did not regress. Compared to the cases with LNG-IUD treatment, the odds ratio of regression of endometrial hyperplasia in untreated cases was 0.013 (95% CI: 0.004, 0.0057, p < 0.0001). Compared to the cases with oral progestogen treatment, the odds ratio of regression of endometrial hyperplasia in untreated cases was 0.098 (95% CI: 0.0359, 0.2798, p < 0.0001).

**Figure 3:**
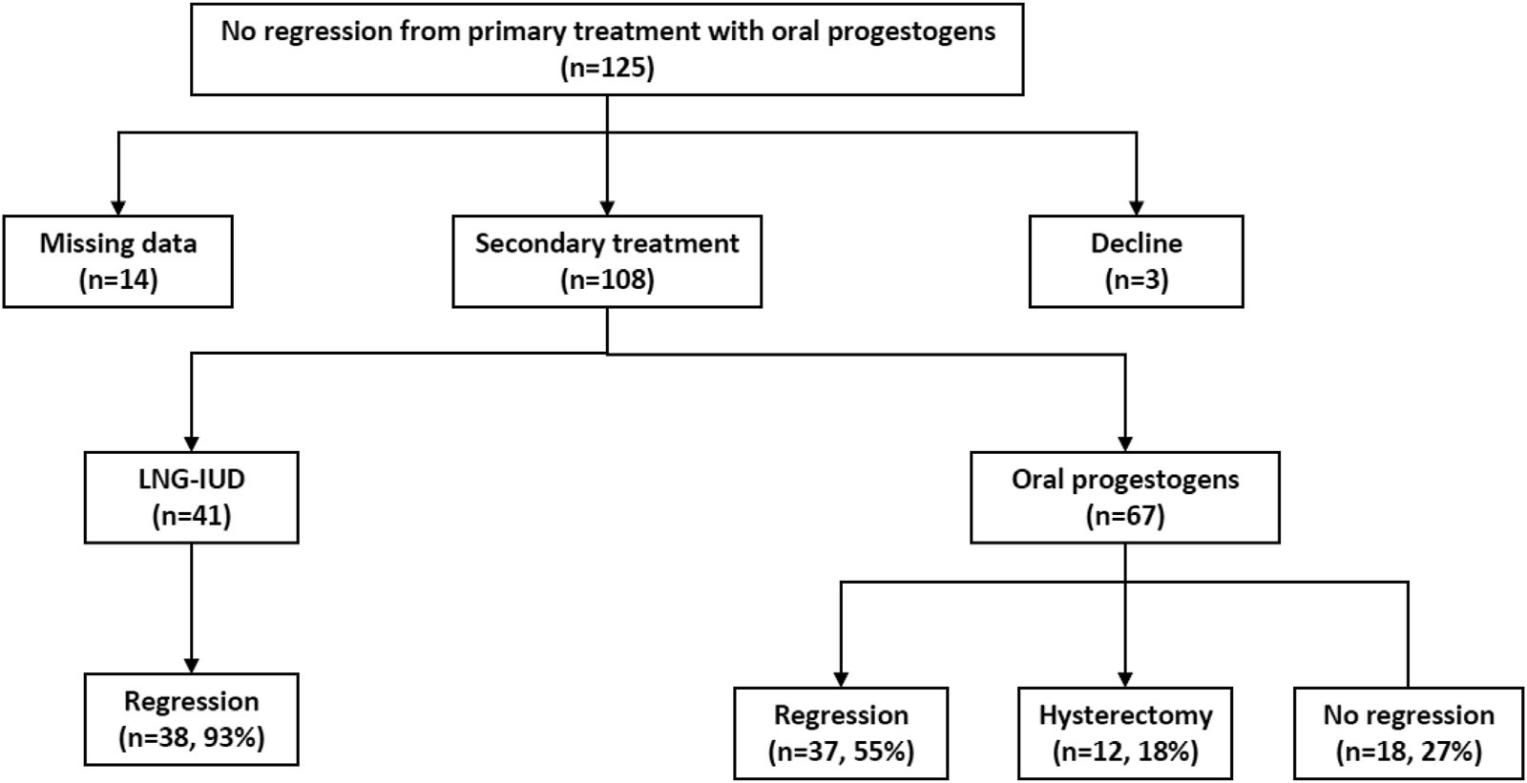
Clinical outcomes in the secondary treatment in cases without regression by primary oral progestogen treatment.

We then analysed the outcomes of the secondary treatment in cases who did not regress after the primary treatment with oral progestogens (n = 108, as 3 cases declined for the secondary treatment and there were missing data in 14 cases) (Figure 3). Of the 108 cases who received the secondary treatment, 67 cases continuously received oral progestogens and 41 cases then alternatively received treatment with LNG-IUD. The clinical outcomes of the secondary treatment of these cases are summarised in Table 3. 38 (93%) cases who alternatively received LNG-IUD as a secondary treatment had regression of endometrial hyperplasia. While, 37 (55%) cases who continuously received oral progestogens as a secondary treatment had regression, which was significantly lower than that in cases alternatively receiving LNG-IUD treatment (p < 0.0001) (Table 3). The odds ratio of regression of endometrial hyperplasia in cases with LNG-LUS as the option of secondary treatment was 10.27 (95% CI: 3.078, 33.65, p < 0.0001), compared to the cases who continuously received oral progestogens as a secondary treatment (Table 3). In addition, in cases who continuously received oral progestogens as a secondary treatment (n = 67), 12 (18%) cases progressed to either endometrial hyperplasia with atypia or endometrial cancer, resulting in a hysterectomy (Figure 3). Three cases without regression of endometrial hyperplasia in the primary treatment declined for the secondary treatment and all of them did not regress after 6 months.

**Table 3:** Clinical outcomes of the secondary treatments in cases without regression after primary treatment of oral progestogens (n = 108).

	Oral progestogens (n = 67)	LNG-IUD (n = 41)	OR (95% CI)∗	P value
Regression	n = 37 (55%)	n = 38 (93%)	10.27 (3.078, 33.65)	<0.0001
Progression	n = 12 (18%) (Hysterectomy)	n = 0 (0%)	N/A	N/A
No regression	n = 18 (27%)	n = 3 (7%)	N/A	N/A

∗ The odds ratio (OR) was calculated to compare the regression and non-regression and progression between the two treatments.

**Figure 4:**
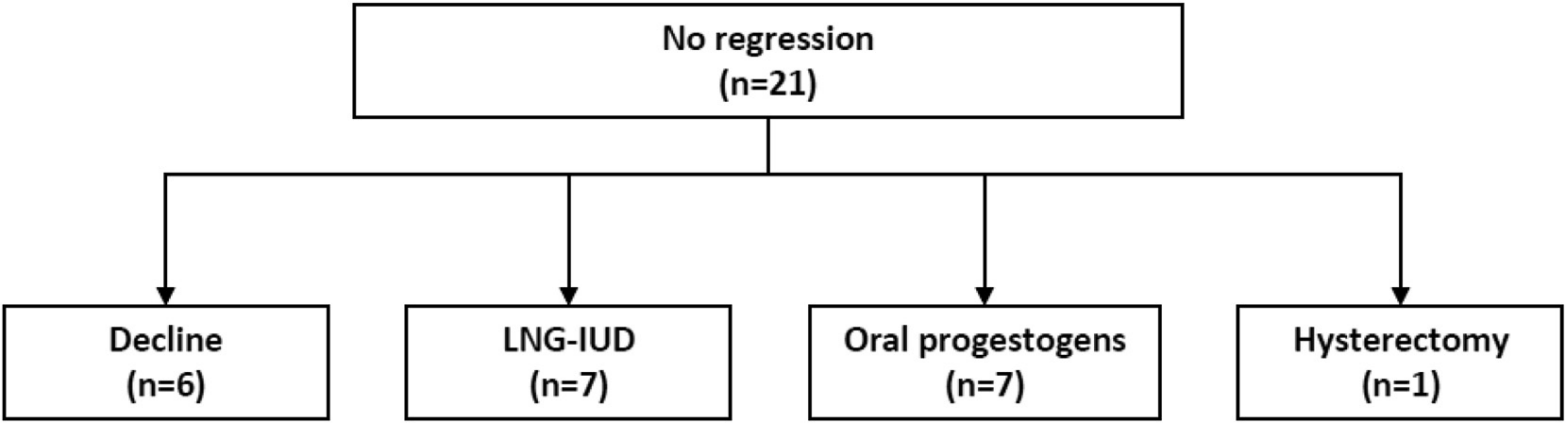
Clinical outcomes in the secondary treatment in untreated cases without regression.

Of the 21 cases who did not receive any primary treatment and did not regress after more than 3 months, one case progressed to endometrial hyperplasia with atypia, resulting in a hysterectomy. Six cases declined further treatment due to personal reasons, and seven cases then received either LNG-IUD or oral progestogens, respectively (Figure 4).

We further analysed the duration of the effectiveness in the regression of endometrial hyperplasia (Table 4). All the cases with oral progestogen treatment who had regression after primary treatment (n = 242) were followed up from 3 to 12 months. Of these 242 cases, 88 (36.4%) cases were further followed up for more than 13 months (up to 68 months). None of these cases had a recurrence of endometrial hyperplasia during the follow-up time. All the cases with LNG-IUD treatment who had regression after primary treatment (n = 69) were also followed up from 3 to 12 months. Of these 69 cases, 30 (40%) cases were further followed up for more than 13 months (up to 76 months). None of these cases had a recurrence of endometrial hyperplasia during the follow-up time.

**Table 4:** The follow-up duration of regression of endometrial hyperplasia in cases received treatment with oral progestogens or LNG-IUD.

	Oral progestogens (n = 242)	LNG-IUD (n = 69)
Short-term follow-up (3–12 months)	154 (63.6%)	39 (56%)
Long-term follow-up (over 12 months)	88 (36.4%)	30 (44%)

## 4 Discussion

In this observational study with relatively large sample size and long-term follow-up, we found that the primary treatment with LNG-IUD showed a 93% regression rate of endometrial hyperplasia, while the primary treatment with oral progestogens showed 66%. The odds ratio of regression of endometrial hyperplasia in cases with LNG-IUS treatment was 7.128 (95% CI: 2.94, 16.76, p < 0.0001), compared to the cases with oral progestogen treatment. Time to regression was significantly shorter in LNG-IUD treated group, compared to oral progestogen treatment group. In addition, in cases without regression by oral progestogens who then received the secondary treatment, the alternative treatment option by LNG-IUD also showed a 93% regression rate of endometrial hyperplasia. While continuously receiving oral progestogen showed a 55% regression rate, which was significantly lower than LNG-IUD treatment as an alternative option. We further found that only a small proportion of untreated cases (16%) regressed from endometrial hyperplasia.

Women with endometrial hyperplasia without atypia traditionally receive hormone progesterone (high-dose oral progestogens) treatment, or intramuscular medroxyprogesterone acetate taken continuously for six months [17, 18]. However, oral progestogens are not always successful at causing regression of endometrial hyperplasia due to there is 12%–53% resistance rate [19], and cyclic progestogens had less effectiveness in the delivery of progestogen on the endometrium than LNG-IUD [20]. Consequently, LNG-IUG being an alternative treatment in women with endometrial hyperplasia without atypia has been commonly used for decades [21]. A systematic review including 24 low-quality studies reported that LNG-IUD treatment reached a 92% pooled regression rate for complex endometrial hyperplasia, which was significantly higher than oral progestogens (66%) [15]. However, this study found no difference in the regression rate for simple endometrial hyperplasia between LNG-IUD and oral progestogen treatment [15]. Another observational study also reported a 94.8% of regression rate in women with non-atypical or atypical endometrial hyperplasia treated with LNG-IUD, compared to an 84% regression rate in women treated by oral progestogens [22]. A recent Cochrane study with 11 RCTs reported an 85%–92% regression rate of non-atypical endometrial hyperplasia treated by LNG-IUD, in contrast, treatment with oral progestogens reached a 72% regression rate with moderate-quality evidence [6]. In comparison with these studies, our current study found a 93% regression rate in women with non-atypical endometrial hyperplasia treated by LNG-IUD, compared to a 66% regression rate by oral progestogens. Collectively, in agreement with other studies, our data show a significantly better outcome in women with non-atypical endometrial hyperplasia by treatment with LNG-IUD. In addition, we found that time to regression was statistically significantly shorter in LNG-IUD treatment group, compared to the progestogen treatment group. This further suggested that the effectiveness of LNG-IUD in the regression of endometrial hyperplasia may be better than oral progestogens.

It has been reported that significant responses in regression of endometrial hyperplasia are commonly seen in 3–6 months after oral progestogen treatment [23]. To date, most studies were performed within 12 months, and the long-term (13 months to two years) effectiveness of regression was of low-quality evidence [6]. A study suggested that the LNG-IUSD effectively opposes the estrogenic effect on the endometrium in the absence of systemic effects [20]. In our current study, 44% of cases treated by LNG-IUD who regressed were followed up for more than 13 months, up to 68 or 76 months, and none of these cases had a recurrence of endometrial hyperplasia during the study period. Our study suggests that there is long-term effectiveness in the regression of endometrial hyperplasia after being treated with LNG-IUD.

There was no study reporting the secondary treatment option in women with endometrial hyperplasia who failed primary treatment with oral progestogens. In our current study, we further investigate the effectiveness of LNG-IUD, as an alternative option for endometrial hyperplasia in cases who did not regress by oral progestogen treatment. We found a 93% regression rate of endometrial hyperplasia in women without regression in their primary treatment by oral progestogens, when they changed treatment to LNG-IUD. In contrast, continuous treatment with oral progestogens only showed a 55% regression rate of endometrial hyperplasia in those cases. In addition, there were 12 (18%) cases resulting in hysterectomy in cases who continuously received oral progestogens, however, there was no hysterectomy in cases that changed to treatment with LNG-IUD in their secondary treatment. Our data support previous studies showing a lower hysterectomy rate in women with LNG-IUD treatment [24, 25, 26].

The condition of endometrial hyperplasia without atypia may improve without treatment, as this type of endometrial hyperplasia is unlikely to progress to cancer. However, to date, only one RCT study with moderate-quality evidence showed a lower regression (15%) of endometrial hyperplasia without atypia in cases without treatment [27]. In our current study, we found that the regression rate was 16% in untreated cases. Compared with LNG-IUD or oral progestogen treatment, the odds ratio of regression of endometrial hyperplasia in untreated cases was 0.013 (95%CI:0.004, 0.0057, p < 0.0001), or 0.098 (95%CI: 0.0359, 0.2798, p < 0.0001). Our data further supported that the regression of endometrial hyperplasia without atypia in the non-treatment group is extremely low.

The strengths of this observational study are that 1) this study included a large sample size; 2) our data showed long-term effectiveness of LNG-IUD; 3) we further analysed the regression rate in cases who failed primary treatment with oral progestogens and then received the LNG-IUD as an alternative option. However, there are also some limitations in this study. The failure of oral progestogen treatment may depend on various reasons such as the age of onset, menopause status, BMI, and healthy condition. In our current study, we found 34% failure rate by oral progestogen treatment. Due to the data availability, we do not know the causes of no response to oral progestogens in these cases. However, it has been reported a 12%–53% resistance to progestogen treatment [19]. Future studies considering lifestyle changes such as weight gain or loss should be performed. Secondly, there was no data on the status of menopause. We cannot conclude whether the regression rate is associated with the status of menopause. However, the age at diagnosis was not a complete surrogate for menopause, as the majority of the cases (80%) were under 50 years. The peak incidence of endometrial hyperplasia without atypia was in the early 50s [1]. Thirdly, the treatment option for individual cases was dependent on the experiences of individual gynaecologists, although there was no intention to treat. Future RCT is required to confirm our findings.

In conclusion, in this observational study with relatively large sample size, we found significant long-term better responses on regression of endometrial hyperplasia after LNG-IUD treatment, in comparison with oral progestogen treatment. In addition, this well response on regression of endometrial hyperplasia was also seen in women who failed with oral progestogens as a primary treatment and then changed treatment to LNG-IUD.

## Declarations

### Author contribution statement

Ye Shen, Hua Fang, Yan Du and Rong Cai: Performed the experiments.

Yi Zhang: Analyzed and interpreted the data.

Min Zhao: Conceived and designed the experiments; Wrote the paper.

Qi Chen: Conceived and designed the experiments; Analyzed and interpreted the data; Wrote the paper.

### Funding statement

This study was supported by the 3rd round of Key Disciplines of Maternal and Child Health in 10.13039/501100002949Jiangsu Province China (reference number: SFY3-JS2021) and The General Scientific Research Project of Wuxi Municipal Health Commission (reference number:M202057).

### Data availability statement

Data will be made available on request.

### Declaration of interest's statement

The authors declare no conflict of interest.

### Additional information

No additional information is available for this paper.
